# Dietary Iron Intake in Women of Reproductive Age in Europe: A Review of 49 Studies from 29 Countries in the Period 1993–2015

**DOI:** 10.1155/2019/7631306

**Published:** 2019-06-13

**Authors:** Nils Thorm Milman

**Affiliations:** Department of Clinical Biochemistry, Næstved Hospital, University College Zealand, DK-4700 Næstved, Denmark

## Abstract

**Objective:**

Assessment of dietary iron intake in women of reproductive age in Europe.

**Design:**

Review.

**Setting:**

Literature search of dietary surveys reporting intake of iron using PubMed, Internet browsers, and national nutrient databases in the period 1993–2015.

**Subjects:**

Women of reproductive age.

**Results:**

49 dietary surveys/studies in 29 European countries were included. Belgium, Bosnia, Denmark, Hungary, Italy, Northern Ireland, Serbia, Scotland, Sweden, Switzerland, United Kingdom/England, and Wales reported a median/mean iron intake of 7.6–9.9 mg/day. Finland, Iceland, Ireland, the Netherlands, Norway, Poland, and Spain reported an intake of 10.0–10.7 mg/day. Austria, Estonia, France, and Russia reported an intake of 11.0–11.9 mg/day. Latvia and Germany reported an intake of 12.0–12.2 mg/day. Croatia, Lithuania, Portugal, and Slovakia reported an intake of 15.9–19.0 mg/day. The percentage of dietary iron consisting of heme iron, reported in 7 studies, varied from 4.3% in United Kingdom to 25% in Spain. Nutrient density for iron (mg iron/10 MJ, median/mean) varied from 11.8 in Sweden to 23.0 in Lithuania. The correlation between nutrient density and dietary iron was significant (*p*=0.0006). In most countries, the majority of women had a dietary iron intake below 15 mg/day. In Belgium, Denmark, Hungary, and Sweden, 91–95% of women had an intake below 15 mg/day. In Ireland and Germany, 61–78% had an intake below 15 mg/day.

**Conclusions:**

In Europe, 61–97% of women have a dietary iron intake below 15 mg/day. This contributes to a low iron status in many women. We need common European standardized dietary methods, uniform dietary reference values, and uniform statistical methods to perform intercountry comparisons.

## 1. Introduction

In healthy humans, the body iron input is generated by gastrointestinal absorption of dietary iron [1]. Body iron losses are composed of basal (obligatory) losses, which are quite similar in men and women [1]. In addition, women in the reproductive age have considerable physiological iron losses associated with menstruations [2, 3] and pregnancies [4]. Both iron deficiency [5] and iron overload [6] will affect body functions in negative ways and impair quality of life and survival.

The World Health Organization's (WHO) latest report on the global prevalence of anemia [7] states that, in the European Region, among nonpregnant women of 15–49 years of age, 22.5% have anemia, predominantly due to iron deficiency (ID).

The high physiological iron losses in women of reproductive age pose demands on iron absorption, which in turn is dependent on the quantitative and qualitative intake of dietary iron. A recent review of body iron status in women of reproductive age in Europe [8] showed that approximately 40–55% have small or absent body iron reserves, i.e., serum ferritin ≤30 *μ*g/L. The prevalence of ID was 10–32% and of iron deficiency anemia (IDA) 2–5%. Approximately 20–35% of women had sufficient iron reserves (serum ferritin >70 *μ*g/L) which could enable them to complete a pregnancy without taking supplementary iron [8]. The low iron status in the majority of European women may in part be due to an inadequate dietary iron intake, a low intake of heme iron, and/or an inappropriate balance between the content of enhancers and inhibitors of iron absorption in the diet.

The objective of this paper was to provide a review of surveys and studies assessing dietary iron intake in nonpregnant women of reproductive age in Europe and to address to which degree dietary iron intake corresponds to the recommended intake in the respective countries.

## 2. Methods

We conducted literature searches in PubMed and on the Internet using the browsers Google Chrome, Google Scholar, and Edge applying the key words “dietary iron intake,” “dietary iron intake in… name of country,” and “dietary survey in… name of country.” Search was performed for all the European countries. We included studies from The European Nutrition and Health Report 2009 [9] and studies from The European Food Safety Authority (EFSA) paper on iron 2015 [10] as well as in the EFSA paper on Dietary Reference Values 2017 [11]. We consulted the EFSA food composition database but could not obtain access to the data on micronutrients. Some studies were identified from references in original papers.

We consulted a paper about information on European national dietary surveys [12] and a paper on European food databases [13]. Several national institutes of nutrition were contacted to obtain information about dietary iron intake. We located no studies from Slovenia and Cyprus and could not obtain information from Greece and North Macedonia and from the Eastern European countries Belarus, Bulgaria, Czech Republic, Moldova, Romania, and Ukraine.

Only surveys/studies reporting the intake of dietary iron *per se* were included in this review. Five studies, which reported the total iron intake, e.g., dietary plus supplemental iron intake but did not specify the dietary iron intake *per se*, were not included in this review.

In the statistical interpretation of the results, it is important to consider the overall distribution of dietary iron intake when evaluating the prevalence of an inadequate iron intake. If the distribution is normal, it is relatively simple to calculate and define inadequacy using parametric statistics with arithmetic mean and standard deviation. In case of an asymmetric distribution skewed to the right, with a higher frequency of low values, the median is lower than the arithmetic mean. Therefore, using the arithmetic mean in skewed data will tend to underestimate the prevalence of inadequacy, and nonparametric statistics should be used instead.

## 3. Results

Most reports were in English language, but we managed to interpret reports published in Dutch, Finnish, French, German, Hungarian, Icelandic, and Spanish languages. We decided to include all the identified reports, irrespective of the dietary method used, in order to demonstrate some of the differences between the dietary methods.

An overview of the 49 included European surveys/studies [14–58] on dietary iron intake in women of predominantly reproductive age performed in 29 countries during the period 1993 to 2015 is shown in Table 1.

In Europe, the age interval for assessing iron intake in women of reproductive age should probably be 20–45 years; on the average, in 2015, across the European Union (EU), the mean age of women was 29 years when they became mothers for the first time and the proportion of teenage mothers less than 20-year-old was on the average 4%, being lowest in the Western EU countries [59]. After the age of 45 years, fertility is markedly reduced; the median age at menopause among white women from industrialized countries ranges between 50 and 52 years and the onset of the perimenopause is 47.5 years [60]. However, many studies used an age interval of 18–64 years, and we had no opportunity to recalculate the results in the 20–45 years interval. However, due to the relative age composition of the women in the respective population groups, the majority was in the reproductive (premenopausal) age, i.e., 20–45 years of age. Furthermore, dietary iron intake was not significantly different in premenopausal and postmenopausal women up to the age of approximately 64 years. This was apparent in studies from Denmark [21, 22], the Baltic Republics [23], Finland [24], France [26], the Netherlands [38, 39], Scotland [46], Serbia [47], United Kingdom (UK) [56, 57], and Wales [58]. Therefore, we decided to include the entire group of women aged 18–64 years. For reasons of comparison, values for dietary iron intake in postmenopausal women are also shown in Table 1. Evidently, dietary iron intake was quite similar in premenopausal women and women in the early postmenopausal years.

The dietary survey method varied between the studies (Table 1). The most frequent method was food diary registration for 2–7 days. The second most used dietary method was 24-hour dietary recall. A single 24-hour dietary recall was used in 11 studies and 24-hour dietary recall* × * 2-3 in 11 studies. Four studies used Food Frequency Questionnaires (FFQ), and three German studies used a dietary history interview.

The food composition tables used to calculate dietary iron intake were in most countries based on national nutrient databases. The Baltic Republics used Russian-based food composition tables from the early 1980s and some of the East European countries used USA-based food composition tables, which were adapted for national use.

In the statistical handling of the data, most of the studies used unweighted population data, while 12 studies used weighted data in order to adjust the study sample for the age and gender composition of the entire population in the country.

Dietary heme iron intake was reported in 7 studies (Table 1). The mean percentage of dietary iron intake consisting of heme iron displayed great variation from 4.3% in UK, 5.7% in Belgium, 10.3% in Russia, 10.4% in France, 14.5% in the Netherlands to 20.1%, and 29.7% in Spain.

Nutrient density is the content of a food component per unit of energy. Nutrient density for iron in mg iron per 10 MJ was reported from 9 countries, as shown in Table 2. The median or mean nutrient density varied from 11.8 in Sweden to 23.0 mg iron/10 MJ in Lithuania. Using the data from the various countries, we found a significant correlation between nutrient density for iron and dietary iron intake, as shown in Table 2 (Spearman's *rho* = 0.91, *p*=0.0006).

Table 3 includes the latest and largest studies from the 29 countries arranged according to the magnitude of median or mean dietary iron intake.

There were considerable differences between the reported median or mean iron dietary intake in different countries. Twelve countries (Bosnia, Belgium, Sweden, Northern Ireland, Scotland, Wales, Serbia, UK including England, Denmark, Switzerland, Hungary, and Italy) reported median or mean iron intake ranging from 7.6 to 9.9 mg/day. Norway, Iceland, Finland, the Netherlands, Spain, Poland, and Ireland reported iron intake from 10.0 to 10.7 mg/day. Estonia, Russia, France, and Austria reported iron intake from 11.0 to 11.9 mg/day. Latvia and Germany reported intake of 12.0 and 12.2 mg/day, while Portugal, Croatia, Slovakia, and Lithuania reported intake from 15.9 to 19.0 mg/day. The “estimated” median value of the reported median or mean dietary iron intake in all the countries shown in Table 3 was 10.2 mg/day.

The estimated average requirement (AR) is the level of daily nutrient intake that is adequate for half of the people in a population group giving a normal distribution of requirement [61]. Using an estimated AR of 7 mg/day for dietary iron intake in women of reproductive age (Table 4), more than 50% of women in all countries had an intake above AR, in some countries up to 70–100% (Table 3). Applying an AR of 10 mg/day (Table 4), more than 50% of women in 13 countries had an intake below AR; in one country, the intake was equal to AR, while in 15 countries, more than 50% of women had an intake above AR.

In the various studies, there was no consistence in the terminology and the use of Dietary Reference Values (DRV) for dietary iron intake. DRVs comprised recommended intake (RI), reference nutrient intake (RNI), recommended dietary allowance (RDA), dietary reference intake (DRI), or lowest recommended nutrient intake (LNRI). Only few studies reported the AR and none used the term “population reference intake” (PRI) as proposed by EFSA [61]. Many papers referred to national RI/RNI/RDAs but without quoting the actual values. There was a tendency that older studies recommended higher RI/RNI/RDA's values above 15 mg/day than more recent studies, which showed increasing consensus towards a value of 14.8–16.0 mg/day. However, the daily recommended intake of dietary iron by the national nutrition boards in women of reproductive age was quite similar in most countries (Table 1). Most studies used an RI/RNI/RDA of 14.8–15.0 mg/day. In Bosnia, Croatia, France, and Spain, RI/RDA/DRI was 18 mg/day. Two Belgian studies from 2004 to 2006 quoted an RNI of 20 mg/day, whereas the latest study from 2014 quoted an RNI of 15 mg/day.

Table 1 shows the studies which tried to assess the percentage of women having dietary iron intake below the national recommended intake. However, there was no consistency concerning the mode of reporting data, so it is not possible to make reliable comparisons of the estimates in the various countries.

From a statistical point of view, a median or mean iron intake below the RI indicates that more than 50% of the women have an iron intake below RI. In all studies, except the Lithuanian, median or mean iron intake was below an RI of 15 mg iron/day.

## 4. Discussion

In the comparison between countries, several confounders should be considered. The dietary methods were different in many of the studies, and they were not standardized and therefore not directly comparable. The European Food Consumption Survey Method (EFCOSUM) group concluded that “the most suitable method to get internationally comparable new data on population means and distributions of actual intake is 24-hour recall, to be conducted at least twice” [66].

The FFQ tend to estimate higher mean intakes for most nutrients compared to the 24-hour recall [67]. In the two Swedish surveys, the regional FFQ study from Umeå [52] reported a mean dietary iron intake of 14.5 mg/day, while the nationwide 4 days food diary study [53] reported a significantly lower intake of 9.4 mg/day. In Croatia, the nationwide FFQ survey [20] reported a mean iron intake of 16.1 mg/day, while a study in nearby Bosnia [19] using the 24-hour recall reported a significantly lower mean iron intake of 7.6 mg/day. Clearly, FFQ should not be used for assessment of dietary iron intake.

Calculation of the dietary intake of micronutrients including iron is dependent on the quality and representativeness of the food composition tables. The food composition tables used to calculate dietary iron intake were in most countries based on national food databases, and the food composition could therefore vary between countries due to the different chemical methodologies used in the analyses of the various food items. For example, the Baltic Republics used old Russian food composition tables and some eastern European countries used food databases from the USA, which were “adapted to regional conditions.”

Food composition tables reflect the composition of the most common staple foods available in a specific country. Usually, mandatory fortification of foods is included in the food composition tables, whereas optional fortification is not. There might in some countries exist foods which are iron-fortified on a voluntary basis, and this iron may not be included in the food composition tables. Iron-fortified foods will contribute to a higher iron nutrient density and consequently to a higher dietary iron intake. This could in part explain the differences in dietary iron intake across Europe.

Countries have different recommendations concerning iron fortification of foods. For example, UK has mandatory fortification of wheat flour (apart from wholemeal) with iron and all flours (except wholemeal) with calcium—a peculiar combination—as calcium is an inhibitor of iron absorption. Many breakfast cereals are fortified with iron on an optional basis and according to the British National Diet and Nutrition Survey, they contribute to 20% of the average iron intake of British adults. Fortification practices in Europe around 2006 were as follows: Denmark, Finland, Germany, Ireland, Italy, the Netherlands, and Spain had no mandatory iron fortification. Denmark has optional fortification with iron to certain flours and breakfast cereals, and fortification of breakfast cereals is also practiced in some other countries [68].

As seen in Table 1, the dietary iron intake expressed as the median was consistently lower than that expressed as the arithmetic mean. This indicates than iron intake in the female population does not follow a normal distribution. The distribution is skewed to the right just like the distribution of menstrual blood losses [2], the distribution of requirements for absorbed iron [69], and the distribution of the iron status biomarker serum ferritin [70]. Therefore, the distribution of iron intake would be better described with nonparametric statistics as median and 2.5–97.5 or 5–95 percentiles. Alternatively, geometric mean and standard deviation can be used. Geometric mean ± standard deviation is calculated as the mean ± standard deviation of log_10_ values of iron intake. None of the studies used geometric mean values. The inconsistency of the used statistical methods impedes direct comparison of the results of different studies.

In general, the national dietary surveys did not correct for underreporting or overreporting of dietary energy intake [71]. Underreporting is assumed to be present in 20–30% of the participants, especially in women and obese persons [72]. Underreporting means that the lowest percentiles for dietary iron intake in women of reproductive age should be taken with considerable reservation. Underreporting is a confounding factor and a major limitation of self-reported dietary intake [72]. Various levels of underreporting in the different studies may contribute to the different results concerning dietary iron intake. Correcting for underreporting will push the population mean and median intakes upwards but could be necessary to get a more accurate picture of intakes in a population.

The variations in dietary iron intake are the resultant of different factors, of which the nutrient density for iron is important. Dietary iron intake is closely associated with nutrient density for iron (Table 3). The nutrient density for iron is dependent on the dietary habits, which differ from country to country, especially concerning the intake of meat, poultry, and fish, which contain easily absorbed heme iron vs. intake of foods containing nonheme iron, which has a lower absorption rate. Nutrient density can also be influenced by mandatory or optional fortification of various food items.

There were marked differences between median and mean dietary iron intake in the various countries, ranging from 7.6 mg/day in Bosnia to 19 mg/day in Lithuania. In almost all countries, more than half of the women of reproductive age had a dietary iron intake below 15 mg/day, ranging from 61% in Ireland to 90–97% in Belgium, Denmark, Hungary, Serbia, and Sweden (Table 1). However, we found no consistent differences between dietary iron intake across the different European regions (western, middle, and eastern Europe) as also previously reported by Elmadfa [9].

The recommended intake for dietary iron was different in many countries, especially before the year 2000 (Table 1). Hereafter, there seems to be growing consensus about the RI, probably encouraged by the recommendations by national and international committees, e.g., EFSA [10] and the Nordic Nutrition Recommendations (NNR) [64]. In most studies after the year 2000, an RI/RNI/RDA for dietary iron intake of 14.8–16 mg/day is recommended. The recommendations for dietary iron intake from different institutions in Europe and the USA [10, 11, 62–65] are listed in Table 4. According to the most recent European recommendations for dietary iron intake in women from the Scientific Advisory Committee on Nutrition in UK (SACN), NNF, and EFSA ranging from 14.8 to 16.0 mg/day (Table 4), we have chosen to use an RNI for iron of 15.0 mg/day in this review.

RI/RNI/RDA is used for assessment of the individual's intake, so comparing median or mean daily dietary iron intake in a female population group against the RI could overestimate the fraction of women who does not obtain their iron requirements. The estimated AR is the daily level of intake that is adequate for 50% of the individuals in a population group [11, 61]. From this aspect, AR is an important DRV in population surveys [11]. If the median or mean iron intake is close to the AR in a female population group, then at least 50% of the women will be meeting their requirements. The higher above the AR the intake is, the greater the proportion of women that should be meeting their requirements. This assumption is valid in populations where nutrient requirements display a normal distribution. However, in women of reproductive age, individual iron requirements show great variation due the variation in menstrual blood losses [3]. Both the distributions of menstrual blood losses [2] and individual iron requirements are skewed, wherefore the estimation of AR is associated with some uncertainty. In Europe, the AR for iron in women of reproductive age is estimated to be 7 mg according to EFSA [10, 11] and 10 mg according to NNR [64]. In the USA, the AR is 8.1 mg [62]. However, few studies reported the estimated AR for dietary iron, and only three studies reported the percentage of women having an iron intake below AR.

In 18- to 45-year-old Danish women, median dietary iron intake was 9.7 mg/day [22]. Using an AR of 7 mg/day, 13% had an intake below AR and 87% an intake equal to or above AR; from these values, it should be anticipated that most women would have an adequate iron status. Using an AR of 10 mg/day, 54% had an intake below AR and 46% an intake equal to and above AR; this is more consistent with the fact that approximately 45% of Danish women of reproductive age have a low iron status with small or depleted body iron reserves [3, 4, 70]. Therefore, in a Scandinavian female population of reproductive age, it seems more appropriate to use an AR of 10 mg/day than an AR of 7 mg/day.

The discrepancies between the RI and actual dietary iron intake may be due to (1) a low nutrient density of iron in the habitual daily diet indicating (2) a low intake of heme iron contained in meat, poultry, and fish [73] and (3) a high intake of ferric nonheme iron from nonanimal foods. Other factors, which influence the absorption of the ingested dietary iron, are a relatively low intake of promotors of iron absorption including the so called “meat factors” which also enhance absorption of nonheme iron [74] and a relatively high intake of inhibitors of iron absorption such as polyphenols, phytates, and calcium. A low energy intake due to the sedentary lifestyle in the affluent western societies may also contribute to a low dietary iron intake but is difficult to balance against the increasing frequency of obesity.

The low dietary iron intake and/or the low iron bioavailability in women in Europe is reflected in their low body iron status. An overview of iron status in women of reproductive age in Europe has recently been published [8] based on data from more than 15 European countries including national surveys and relevant clinical studies. Iron status was assessed by measurement of plasma or serum ferritin and blood haemoglobin concentrations. In women of reproductive age, median or geometric mean serum ferritin concentrations were estimated at 26–38 *μ*g/L. Approximately 40–55% of this population had small or depleted body iron reserves and 45–60% had “replete” iron reserves (serum ferritin >30 *μ*g/L, corresponding to iron reserves of >230 mg). The prevalence of ID (serum ferritin <12–15 *μ*g/L) was 10–32%, and the prevalence of IDA (serum ferritin <12–15 *μ*g/L and haemoglobin <120 g/L) was 2–5%. Only 20–35% of premenopausal women had sufficient iron reserves (serum ferritin >70 *μ*g/L) to complete a normal pregnancy without needing supplementary iron [75].

## 5. Limitations of This Review

This review has several limitations, due to the heterogeneous methods used in the studies. Some studies were national and some regional, the age groups and the dietary methods differed between studies, and statistical methods were different—some studies used nonparametric and others parametric statistics; most studies used unweighted and some weighted data, and few studies had been corrected for underreporting; furthermore, there was an inconsistent terminology concerning the use of DRVs of dietary iron. These are all factors, which impair comparison of the results of the various studies. Furthermore, the food composition tables varied from country to country, and the contribution of voluntary food iron fortification was not evaluated in the studies.

## 6. Conclusions

This review demonstrates that, in Europe, a high proportion of women of reproductive age have a dietary iron intake below 15 mg/day. The relatively low iron intake may contribute to the low body iron status found in many women in Europe [8].

In European countries and within the European Union, there is a definite need for development and implementation of common standardized dietary methods [66] and for standardization of food composition tables as recently introduced by EFSA [76]. It is also important to obtain consensus on the use of the different DRVs [61] and to implement the use of uniform statistical methods in order to obtain reliable intercountry comparisons of dietary intakes of macro- and micronutrients.

## Figures and Tables

**Table 1:** Dietary iron intake in nonpregnant women of predominantly reproductive age in 29 European countries. For comparison, in some countries, iron intake in postmenopausal women is shown as well.

Country and name of survey (if not nationwide, the region is quoted)	Study period	Women, *n*	Age, yrs range	Dietary method	Dietary iron intake (mg/day)				Weighted data	Heme iron, mean % of iron intake	Dietary iron density, mg/10 MJ	Recommended iron intake in women^9^ at time of study (mg/day)	Iron intake below recommended % of women^9^	Reference
					Mean ± SD^2^	Median	Percentiles/CI^4^	IQ^8^ range						
Austria (Österreichischer Ernährungsbericht)	2007	1345	19–64	24-hour recall	11.9 ± 4.4	—	—	—	No	—	—	RI 15	Mean intake >15% lower than RI	[14]
Austria (Österreichischer Ernährungsbericht)	2011	180	18–50	24-hour recall × 2	∼11.0	—	∼10.2–12.0^4^	—	Yes	—	—	RI 15	—	[15]
Belgium (De Belgische Voedselconsumptiepeiling)	2004	460	19–59	24-hour recall × 2	9.9 ± 2.2	9.7	—	8.3–11.3	No	—	—	RNI 20	100% had intake < RNI	[16]
Belgium (City of Ghent)	2006	641	18–39	2 days food diary	—	10.6	—	9.0–12.8	No	5.7	Median 13.1 (11.0–15.3^8^)	RDA 20	Median intake 53% of RDA	[17]
Belgium (De Belgische Voedselconsumptiepeiling)	2014	300	18–39	24-hour recall × 2	8.4	—	8.2–8.9^4^	—	No	—	—	AR 7 RNI 15	96% had intake < RNI	[18]
Bosnia (Canton Una-Sana)	∼2014	∼136	18–50	24-hour recall	7.6 ± 7.9	—	—	—	No	—	—	RDA 18	100% had intake < RDA	[19]
Croatia (University Centers)	∼2005	480	18–30	FFQ^1a^	16.1 ± 7.9	—	—	—	No	—	—	DRI 18	Mean intake significantly lower than DRI	[20]
Denmark (Danskernes Kostvaner)	2003–2008	933	18–45	7 days food diary	9.0 ± 2.5	8.8	5.2–13.2^5^	7.4–10.5	No	—	—	RDA 15	—	[21]
Denmark (Danskernes Kostvaner)	2003–2008	1569	19–64	7 days food diary	9.0 ± 2.4	8.8	5.3–13.2^5^	7.4–10.5	No	—	—	RDA 15	—	[21]
Denmark (Danskernes Kostvaner)	2003–2008	1785	18–75	7 days food diary	9.0 ± 2.5	8.8	6.1–12.0^6^	—	No	—	Mean 11.5; median 11.3 (9.2–14.0^6^)	RDA 15	—	[21]
Denmark (Danskernes Kostvaner)	2011–2013	686	18–45	7 days food diary	9.9 ± 2.9	9.7	5.6–14.5^5^	8.1–11.5	No	—	—	AR 10 RDA 15	54% had intake < AR; 97% had intake < RDA	[22]
Denmark (Danskernes Kostvaner)	2011–2013	1259	19–64	7 days food diary	10.1 ± 2.8	9.9	5.8–14.8^5^	8.2–11.7	No	—	—	RDA 15	—	[22]
Denmark (Danskernes Kostvaner)	2011–2013	1552	18–75	7 days food diary	10.0 ± 2.9	9.8	6.7–13.4^6^	—	No	—	Mean 12.0; median 11.9 (9.6–14.6^6^)	RDA 15	—	[22]
Estonia (WHO Nutrition and Lifestyle in the Baltic Republics)	1997	835	19–49	24-hour recall	12 ± 7	11	—	—	No	—	Mean ∼18 ± 8.0^2^	—	—	[23]
Estonia (WHO Nutrition and Lifestyle in the Baltic Republics)	1997	280	50+	24-hour recall	11 ± 7	10		—	No	—	Mean 18 ± 8.0^2^	—	—	[23]
Finland (FINDIET, City of Helsinki)	1997	118	25–49	24-hour recall	10.3	—	—	—	No	—	Mean 15	RDA 12–18	—	[24]
Finland (FINDIET, City of Helsinki)	1997	67	50–54	24-hour recall	9.1	—	—	—	No	—	Mean 15	RDA 9^10^	—	[24]
Finland (FINDIET)	2012	710	25–64	24-hour recall × 2	10.3 ± 4.0	10.3	6–16^5^	—	No	—	Mean 14 (13–15^4^); 95% interval	AR 10 RDA 15	Mean intake 68% of RDA	[25]
France (SU.VI.MAX)	1995–1996	Fraction of 3111	35–44	6 days food diary	—	11.8	∼7.1–18.7^5^	—	No	10.4	Median 15.8 (11.5–24.4^5^)	RDA 18	93% had intake < RDA	[26]
France (SU.VI.MAX)	1995–1996	Fraction of 3111	45–60	6 days food diary	—	11.8	∼7.0–19.7^5^	—	No	10.9	Median 15.8 (11.5–24.4^5^)	RDA 10	—	[26]
France (INCA1)	1998–1999	536	18–54	7 days food diary	∼11.2 ± 3.0	—	—	—	No	—	—	—	—	[27]
France (INCA2)	2006–2007	657	18–54	7 days food diary	∼11.9 ± 3.8				No	—	—	—	—	[27]
Germany (GENUS)	1998	2267	18–79 (47% < 45 y)	DHI^1b^	∼14.2		∼13.8–14.6^11^		No	—	—	—	—	[28]
Germany (Ernährungsverhalten in Deutschland)	1998	1228	18–44	DHI^1b^	—	∼13.6	∼9.5–19.2^6^		No	—	—	RDA 15	—	[29]
Germany (Ernährungsverhalten in Deutschland)	1998	381	45–54	DHI^1b^	—	∼13.7	∼9.4–19.4^6^		No	—	—	RDA 10	—	[29]
Germany (Nationale Verzehrsstudie II)	2006	4176	19–50	DHI^1b^	∼12.6 ± 0.11^3^	∼12.2	∼7.1–20.0^5^	∼9.8–17.9	No	—	—	RDA 15	∼78% had intake < RDA	[30]
Hungary (OTAP)	2003–2004	471	18–64	3 days food diary	9.7 ± 0.12^3^		9.5–10.0^4^		Yes	—	—	RDA 15	91% (88–93^4^) had intake < RDA	[31]
Hungary (OTAP)	2009	527	18–64	3 days food diary	9.8 ± 0.2^3^		9.5–10.2^4^		Yes	—	—	RDA 15	90% (87–92^4^) had intake < RDA	[32]
Hungary (OTAP)	2014	375	18–64	3 days food diary	9.8 ± 0.2^3^		9.4–10.2^4^		Yes	—	—	RDA 15	87% (82–90^4^) had intake < RDA	[33]
Iceland (Hvað borða Íslendingar?)	2010–2011	∼150	18–45	24-hour recall × 2	10.2 ± 4.3				No	—	—	RDA 15	Mean intake 68% of RDA	[34]
Ireland (NSIFCS)	1997–1999	555	18–50	3 days food diary	∼10.1 ± 3.4	∼9.6	∼5.6–16.3^5^		No	—	—			[35]
Ireland (NANS)	208–2011	640	18–64	4 days food diary	13.7				Yes	—	—	RI 14.8	61% had intake < RI	[36]
Italy (INRAN-SCAI)	2005–2006	1244	18–65	3 days food diary	10.2	9.9	5.7–15.8^5^		No	—	—			[37]
Latvia (WHO Nutrition and Lifestyle in the Baltic Republics)	1997	738	19–49	24-hour recall	13.0 ± 7.0	13			No	—	Mean 18.0 ± 8.0^2^			[23]
Latvia (WHO Nutrition and Lifestyle in the Baltic Republics)	1997	497	50+	24-hour recall	13.0 ± 8.0	12			No	—	Mean 19.0 ± 8.0^2^			[23]
Lithuania (WHO Nutrition and Lifestyle in the Baltic Republics)	1997	752	19–49	24-hour recall	∼20.5 ± 12.5	18.5			No	—	Mean 26.0 ± 13.5^2^			[23]
Lithuania (WHO Nutrition and Lifestyle in the Baltic Republics)	1997	380	50+	24-hour recall	20.0 ± 12.0	18.0			No	—	Mean 24.0 ± 14.0^2^			[23]
Netherlands	1996	111	20–49	3 days food diary	∼11.0 ± 2.8				No	14.5	—	RDA 15	Mean intake ∼29% of RDA	[38]
Netherlands	1996	111	50–79	3 days food diary	∼10.5 ± 2.8				No	16.2	—	RDA 8^10^	Mean intake ∼29% of RDA	[38]
Netherlands (TNO Rapport)	1997–1998	1472	22–50	2 days food diary	10.7 ± 3.2	10.4	6.1–16.2^5^		No	—	—	RDA 15	—	[39]
Netherlands (TNO Rapport)	1997–1998	512	50–65	2 days food diary	10.7 ± 3.3	10.4	5.9–16.7^5^		No	—	—	RDA 8	—	[39]
Netherlands (DNCS)	2007–2010	698	19–50	24-hour recall × 2	—	9.6	6.5–13.5^5^		No	—	—	RDA 15	—	[40]
Northern Ireland (NDNS)	2008–2012	246	19–64	4 days food diary	9.1 ± 2.7	9.1	4.0–14.6^7^		Yes	—	—	RNI 14.8	Mean intake 73% of RNI	[41]
Norway (NORKOST)	2010–2011	—	18–70	24-hour recall × 2	10 ± 4	10	—	7.5–12	No	—	—	AR 10 RDA 15	—	[42]
Poland (Household Food Consumption in Poland)	2000	1334	19–64	24-hour recall	10.7 ± 6.5	—	—	—	—	—	—	—	—	[43]
Portugal (City of Porto)	2004–2005	249	18–40	FFQ^1a^	—	15.9	—	13.3–18.8	No	—	—	AR 8.1	0% had intake < AR	[44]
Russia (Russian Longitudinal Monitoring survey 1–4)	1993–1994	5161	14–54	24-hour recall	11.8		—	8.5–14.0	No	10.3	—	—	—	[45]
Scotland (NDNS)	2008–2012	219	25–49	4 days food diary	9.6 ± 3.0	9.3	4.6–16.3^7^	—	Yes	—	—	RNI 14.8	Mean intake 65% of RNI	[46]
Scotland (NDNS)	2008–2012	119	50–64	4 days food diary	9.3 ± 3.1	8.9	3.4–15.5^7^	—	Yes	—	—	RNI 8^10^	Mean intake of RNI	[46]
Serbia	2013–2015	501	25–49	24-hour recall × 3	9.4 ± 4.0	—	—	—	No	—	—	AR 8.1 RDA 15	95% had intake < RDA	[47]
Serbia	2013–2015	125	50–65	24-hour recall × 3	10.0 ± 5.7	—	—	—	No	—	—	AR 5 RDA 9^10^	50% had intake < RDA	[47]
Slovakia (Dietary Habits of the Slovak Population)	1995–1999	1211	18–80	24-hour recall	18.6 ± 7.1	—	—	—	No	—	—	—	Mean intake was 124% of RDA	[48]
Spain, Catalonia (ENCAT)	1992–1993	757	18–44	24-hour recall × 2	∼11.8	—	—	—		—	—	—	Mean intake 65% of RNI	[49]
Spain (Madrid University)	1996	130	19–35	7 days food diary	11.1 ± 3.0	—	5.1–16.8^5^	—	No	29.7	Mean 14.1, range 6.2–23.4	RI 18	98% had intake < RI; mean intake 62% of RI	[50]
Spain, Catalonia (ENCAT)	2002–2003	558	18–44	24-hour recall × 2	10.5	—	—	—	—	—	—	—	Mean intake 58% of RNI	[49]
Spain (ANIBES)	2013	996	9–75	3 days food diary	—	9.8	—	7.9–11.9	No	20.1	—	RI 18	∼90% had intake < RI	[51]
Sweden (City of Umeå)	2006–2009	206	30–40	FFQ^1a^	∼14.5	—	∼13.9–15.1^4^				—	AR 10 RDA 15	—	[52]
Sweden (Riksmaten)	2010–2011	449	18–44	4 days food diary	∼9.4 ± 3.3	∼9.0	∼4.6–15.0^5^		No		Mean 12.4 ± 3.6^2^; median 11.8 (3.3–18.4^5^)	AR 10 RDA 15	∼95% had intake < RDA	[53]
Switzerland, Canton Geneva (Bus Santé Geneva)	2009	542	34–74	FFQ^1a^	9.8 ± 3.6	—	—	—	Yes	—	—	—	—	[54]
UK (NDNS)	2000–2001	891	19–64	4 days food diary	10.0	9.6	—	7.3–12.2	Yes	4.3	—	RNI 14.8	27% had intake < LRNI	[55]
UK (NDNS) years 1–2	2008–2009	253	19–64	4 days food diary	—	9.9	—	7.8–12.0	Yes	—	—	RNI 14.8	21% had intake < LRNI	[55]
UK (NDNS) years 1–4	2008–2009 and 2011–2012	1571	19–64	4 days food diary	9.6 ± 3.0	9.5	4.1–15.9^7^	—	Yes	—	—	RNI 14.8	Mean intake 78% of RNI	[56]
UK (NDNS) years 1–4	2008–2009 and 2011–2012	436	65+	4 days food diary	9.4 ± 2.7	9.1	4.9–15.3^7^	—	Yes	—	—	RNI 8.7^10^	Mean intake 109% of RNI	[56]
UK (NDNS) years 5–6	2012–2013 and 2013–2014	592	19–64	4 days food diary	9.4 ± 3.1	9.3	4.1–15.7^7^	—	Yes	—	—	RNI 14.8	27% had intake < LRNI; mean intake 76% of RNI	[57]
UK (NDNS) years 5–6	2012–2013 and 2013–2014	193	65+	4 days food diary	9.3 ± 3.2	8.8	4.3–16.5^7^	—	Yes	—	—	RNI 8.7^10^	Mean intake 106% of RNI	[57]
Wales (NDNS)	2009–2010 and 2012–2013	198	19–64	4 days food diary	9.6 ± 3.2	9.4	4.2–16.8^7^	—	Yes	—	—	RNI 14.8	25% had intake < LRNI; mean intake 79% of RNI	[58]
Wales (NDNS)	2009–2010 and 2012–2013	79	65+	4 days food diary	8.9 ± 3.0	8.7	3.3–17.1^7^	—	Yes	—	—	RNI 8^10^	6% had intake < LRNI; mean intake 102% of RNI	[58]

^1a^FFQ = Food Frequency Questionnaire; ^1b^DHI = dietary history interview; ^2^SD = standard deviation; ^3^SE = standard error; ^4^CI = 95% confidence interval; ^5^5–95 percentiles; ^6^10–90 percentiles; ^7^2.5–97.5 percentiles; ^8^IQ range = interquartile range; ^9^women of reproductive age; ^10^postmenopausal women; ^11^95% confidence interval of the mean; DRI = dietary reference intake, reference value used to plan and assess nutrient intake of healthy women (Institute of Medicine, USA); AR = estimated average requirement, daily intake levels estimated to meet the needs of half of the healthy women, provided a normal distribution of requirement; RDA = recommended dietary allowance, daily intake being sufficient to meet the nutrient requirements of nearly all (97-98%) healthy women; RNI = reference nutrient intake also named “population reference intake,” daily intake being sufficient to meet the nutrient requirements of nearly all (97.5%) healthy women; RI = recommended intake, daily intake being recommended to meet the nutrient requirements of healthy women; LRNI = lowest recommended nutrient intake, the amount of a nutrient or energy, which is sufficient for only a few healthy women.

**Table 2:** Association between nutrient density for iron and dietary iron intake arranged according to the magnitude of nutrient density.

Country	Reference	Dietary iron density (mg/10 MJ)	Dietary iron intake (mg/day)
Lithuania	[23]	23.0^1^	19^2^
Estonia	[23]	17.0^1^	11^1^
Latvia	[23]	17.0^1^	12^2^
France	[26]	15.8^2^	11.8^2^
Spain	[50]	14.1^1^	11.1^1^
Finland	[25]	14.0^1^	10.3^2^
Belgium	[17]	13.1^2^	10.6^2^
Denmark	[22]	11.9^2^	9.8^2^
Sweden	[53]	11.8^2^	9.0^2^

^1^Arithmetic mean; ^2^median; Spearman's *rho* = 0.91, *p*=0.0006.

**Table 3:** Dietary iron intake in nonpregnant women of predominantly reproductive age in 29 European countries, arranged according to median or mean iron intake.

Country	Reference	Median or mean iron intake (mg/day)^*∗*^
Lithuania	[23]	18.5
Slovakia	[48]	18.6^*∗*^
Croatia	[20]	16.1^*∗*^
Portugal	[44]	15.9
Germany	[30]	12.2
Latvia	[23]	13.0
Austria	[14]	11.9^*∗*^
France	[27]	11.9^*∗*^
Russia	[45]	11.8^*∗*^
Estonia	[23]	11.0
Ireland	[36]	10.7
Poland	[43]	10.7^*∗*^
Spain	[49]	10.5^*∗*^
Netherlands	[39]	10.4
Finland	[25]	10.3
Iceland	[34]	10.2^*∗*^
Norway	[42]	10.0
Italy	[37]	9.9
Hungary	[33]	9.8^*∗*^
Switzerland	[54]	9.8^*∗*^
Denmark	[22]	9.7
UK/England	[56]	9.5
Serbia	[47]	9.4^*∗*^
Wales	[58]	9.4
Scotland	[46]	9.3
Northern Ireland	[41]	9.1
Sweden	[53]	9.0
Belgium	[18]	8.4^*∗*^
Bosnia	[19]	7.6^*∗*^

^*∗*^Arithmetic mean.

**Table 4:** Average requirement (AR) and reference nutrient intake (RNI) for dietary iron in women in Europe and the USA.

Institution	Reference	AR (mg/day)	RNI (mg/day)	AR (mg/day)	RNI (mg/day)
Age, years	—	<50	<50	≥50	≥50
IOM 2001	[62]	8.1	18	5	8
FAO/WHO 2001	[63]	—	19.6^*∗*^	—	7.5^*∗*^
NNR 2012	[64]	10	15	6	9
EFSA 2015, 2017	[10, 11]	7	16	6	11
SACN 2017	[65]	—	14.8	—	8.7

^*∗*^Provided 15% iron bioavailability; IOM = Institute of Medicine (USA); FAO = Food and Agriculture Organization of the United Nations; WHO = World Health Organization; NNR = Nordic Nutrition Recommendations; EFSA = European Food Safety Authority; SACN = Scientific Advisory Committee on Nutrition (UK).
